# The usefulness of endotracheal tube twisting in facilitating tube delivery to glottis opening during GlideScope intubation in infants: randomized trial

**DOI:** 10.1038/s41598-020-61321-7

**Published:** 2020-03-10

**Authors:** Jeong Jin Min, Eun Jung Oh, Young Hee Shin, Eunjin Kwon, Ji Seon Jeong

**Affiliations:** 1 Department of Anesthesiology and Pain Medicine, Samsung Medical Center, Sungkyunkwan University School of Medicine, Seoul, South Korea; 2 Department of Anesthesiology and Pain Medicine, Kangwon National University Hospital, Chuncheon, South Korea

**Keywords:** Health care, Medical research

## Abstract

Despite an excellent view of the glottis, technical difficulties with endotracheal tube delivery remains in GlideScope intubation. We evaluated whether a spiral-shape twisted tube can facilitate placement of the tracheal tube tip at the center of glottis opening compared to conventional tube for GlideScope intubation in infants. Eighty-six infants were randomly placed in either the conventional tube group (group C) or the twist tube group (group T). In group T, the shaft of the tube was manually twisted into a loose spiral shape. The primary outcome was the initial center location of the tube tip at the glottis opening, and the secondary outcome was total tube handling time. The initial center location rate of the tube tip at the glottis opening was significantly higher in group T than in group C (88% [38/43] vs. 47% [20/43], P < 0.001). In addition, total tube handling time (sec) was significantly shorter in group T than in group C (15.4 ± 4.7 vs. 18.2 ± 5.3, P = 0.012). In this study, the spiral shape twist tube successfully improved the rate of initial center location of the tube tip at glottis opening and facilitated tube delivery in GlideScope intubation in infants.

## Introduction

In infants and neonates, the patient’s small size is known as an independent risk factor for difficult airway management and complications^1^. Although increased use of angulated videolaryngoscopes such as the GlideScope (GlideScope Cobalt AVL, Verathon Inc., Bothell, USA) provide a better laryngeal view than traditional direct laryngoscopy^2–6^, difficulties remain for inserting the tracheal tube into the laryngeal inlet^6–12^.

In studies of GlideScope intubation, technical difficulty was most likely to be associated with tube delivery to the laryngeal inlet requiring prolonged laryngoscopy time and operator experience^6,11,13^, and this difficulty was more prominent in infants with challenging airways^14^. Despite manipulating the tube tip angle imitating the GlideScope blade shape, the tube tip still tends to be placed posterior or to the right side of the laryngeal inlet^15^ requiring additional handling to place the tube tip at the center of the laryngeal inlet.

Previously, there has been a report of tracheal tube twisting that was successfully used in difficult GlideScope intubation cases^16^. Twisting the tube shaft into a spiral shape seems to provide a sufficient angle that allows the tracheal tube tip to be placed at the center of the laryngeal inlet. Nevertheless, to the best of our knowledge, there have been no randomized trials to determine the usefulness of this novel technique for optimizing tube tip placement at the center of the laryngeal inlet during GlideScope intubation in infants.

Therefore, in this randomized clinical trial, we evaluated whether a new spiral-shape twisted tube could facilitate placement of the tracheal tube tip at the center of the laryngeal inlet compared to the conventional hockey stick-shaped tube for GlideScope intubation in infants.

## Methods

The study protocol was approved by the Institutional Review Board of Samsung Medical Center, Seoul, Korea on 28 August 2018 (SMC *2018-04-112-003, Chairperson Prof. Lee Suk-Koo*) and registered on 2 November 2018 at the Clinical Research Information Service (https://cris.nih.go.kr/, Identifier: KCT*0003317*, Principal investigator: Ji Seon Jeong**)**. Written informed consents were obtained from parents or guardians before participating in the study. All methods were performed in accordance with the relevant guidelines and regulations.

### Patients and randomization

From September 2018 to March 2019, infant patients (12 months old or less) undergoing elective surgical procedures in a single tertiary medical center requiring endotracheal intubation were enrolled. Patients who had an endotracheal tube before surgery or who had a known or suspected oropharyngeal obstructive mass or foreign body were excluded from the study. Patients were also excluded if they needed alternative intubation rather than through the oral cavity.

Patients were randomly assigned to either a hockey stick-shaped conventional tube group (Conventional group, group C) or to a spiral shaped twist tube group (Twist group, group T) using computer-generated numbers found in sealed envelopes according to the endotracheal tube shape used.

### Endotracheal tube preparation

The endotracheal tube (Shiley, Hi-Contour Oral/Nasal Tracheal tube, Covidien, Germany) was prepared according to the group allocation before anesthesia induction by one designated anesthesiologist. As our institutional standard of care, all intubation was performed using a cuffed endotracheal tube, and the size of the tube was determined by the attending anesthesiologist with reference to Khine’s formula^17^. The smallest endotracheal tube used in the study was a 3.0 cuffed endotracheal tube (internal diameter 6.0 mm). The size of the GlideScope blade was determined according to the manufacturer’s recommendations as follows: GlideScope blade size 0 for patients under 1.5 kg in weight, size 1 for patients between 1.5 kg to 3.6 kg, size 2 for patients between 1.8 kg to 10 kg, and size 2.5 for patients between 10 kg to 28 kg.

**Figure 1:**
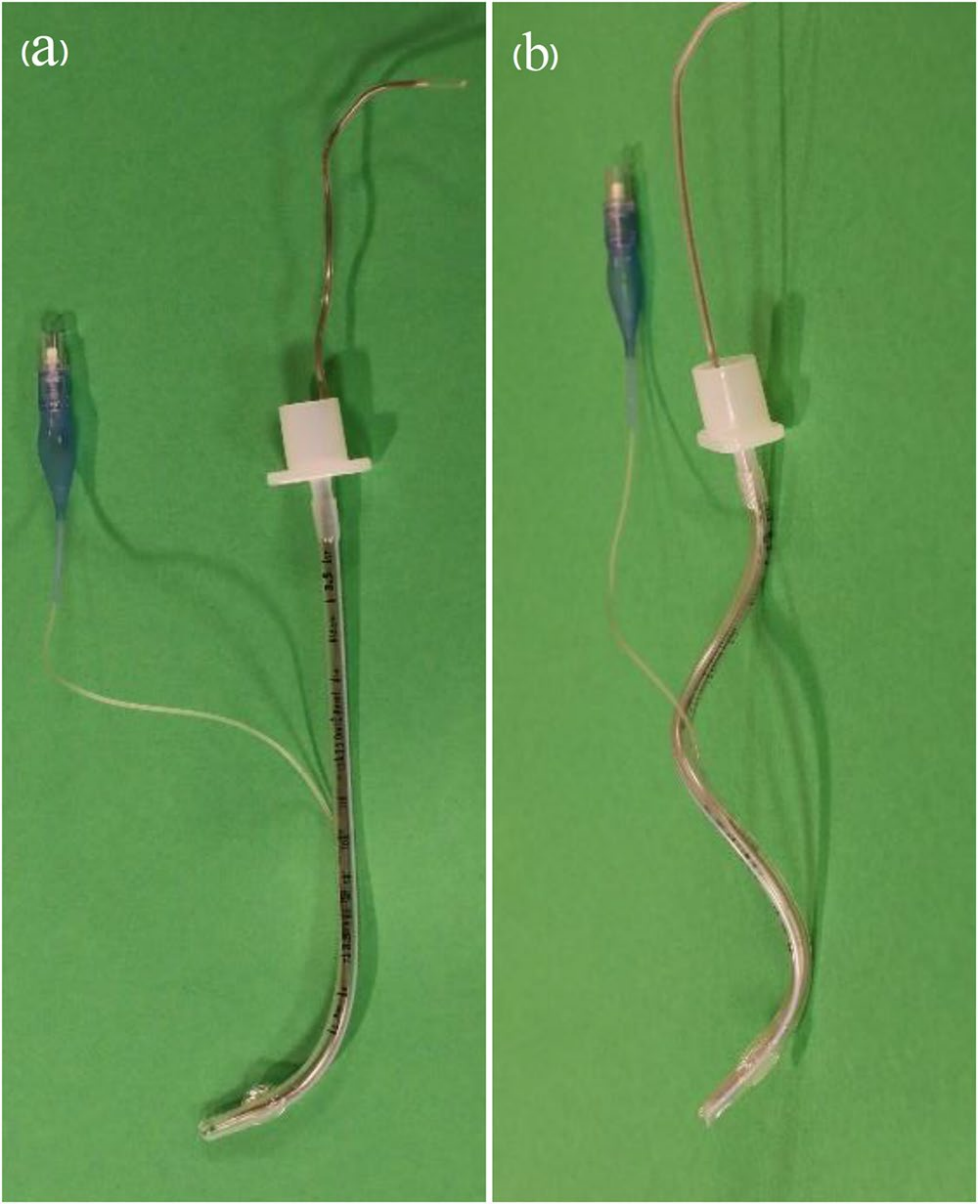
Endotracheal tube molded using a flexible stylet. (**a**) Conventional hockey stick shape tube. (**b**) A new spiral-shape twisted tube.

In the conventional tube group (group C), the shape of the endotracheal tube resembled a hockey stick. The shaft of the tube was left straight as it was manufactured, and only the shape of the tube tip was angled imitating the GlideScope blade using a flexible stylet (6 Fr/Ch. Mallinckrodt Intubating Stylet, Covidien, Athlone, Ireland) (Fig. 1a). This stylet is a single-use flexible device sheathed in polyvinyl chloride (diameter 2.0 mm). In the twist tube group (group T), the shaft of the endotracheal tube was manually twisted into a loose spiral shape. In detail, while holding the connector side of the tube in the right hand and the murphy’s eye side (tip of the endotracheal tube) in the left hand, the connector side was twisted clockwise and the tube tip side was twisted counterclockwise forming a loose spiral shape. The tip of the endotracheal tube was angulated according to the angle of the GlideScope blade as in the conventional group (Fig. 1b).

### Intubation and data acquisition

Patients were not premedicated and routine monitoring for general anesthesia was undertaken. Anesthesia was induced with intravenous thiopental sodium (5–6 mg/kg). Neuromuscular blockade was obtained with intravenous rocuronium bromide (0.6–1.0 mg/kg) to facilitate endotracheal intubation. Manual ventilation was done with facial mask via 3 to 8% sevoflurane in 100% oxygen before laryngoscopy. Patients were intubated using a GlideScope with prepared endotracheal tube shaped according to the group assignment. All intubations were performed by one of two experienced anesthesiologists (J. J. Min and J. S. Jeong) with more than 50 GlideScope intubations in pediatric anesthesia cases. After intubation, the embedded camera in the GlideScope blade was used to identify any soft tissue injuries or other complications related to intubation. The camera was withdrawn to fully visualize the oral cavity. If the patient’s saturation fell below 95% during the intubation process, the intubation was stopped and the patient received mask ventilation with 100% oxygen. During all intubations, all procedures were video recorded using the camera embedded in the GlideScope blade.

**Figure 2:**
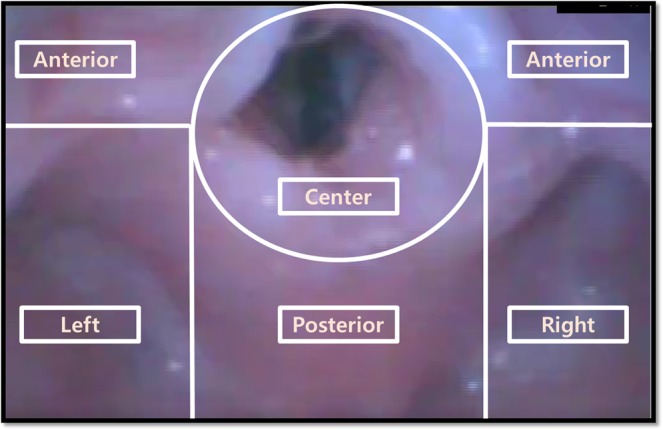
Five sections of the space around the laryngeal inlet.

An independent investigator (E. J. Oh) who was blinded to the study group allocation reviewed all video recordings of the intubation procedures and collected the intubation data for analysis. The video recordings showed the same magnified airway image, which was seen on the GlideScope video monitor screen during the intubation. Videos only showed the tip of the endotracheal tube (from the tube tip to slightly above the balloon) and the shape of the prepared tube shaft was not able to be identified by the assessor. The primary outcome was the initial location of the tube tip at the laryngeal inlet, if it was at the center of the laryngeal inlet or needed an additional re-direction to deliver it to the center. We divided the space around the laryngeal inlet into five sections. “Center” was defined as the endotracheal tip being within a circle drawn along the upper border of the epiglottis to the lower border of cuneiform cartilage and corniculate cartilage (Fig. 2). Then we drew a parallel line between the epiglottis and the arytenoid cartilage outside of the circle. The division above the parallel line was defined as “anterior”, while the space under was divided into three divisions. The division directly below the circle was defined as “posterior”, while division located on either side of the “posterior” division were defined “left” and “right”.

The secondary outcome was total endotracheal tube handling time. The total endotracheal tube handling time measurement started when the GlideScope blade acquired the best view of the laryngeal inlet and ended when the proximal end of the endotracheal tube balloon was advanced through the vocal cords. We divided the total tube handling time into two courses, and the time required for each course was also collected. Course 1 started when the GlideScope blade acquired the best view of the laryngeal inlet and ended when the endotracheal tube tip was placed at the center of laryngeal inlet (tube delivering time). Course 2 started when the endotracheal tube tip passed through the vocal cords and ended when the proximal part of the balloon of the endotracheal tube was fully advanced into the vocal cords withdrawing the stylet at the same time (tube advancement time). Therefore, total tube handling time was the sum of tube delivering time and tube advancement time.

### Statistical analysis

The aim of this study was to compare the initial tube tip location at the center of the laryngeal inlet, and our pilot data showed approximately 30% improvement with the twisted endotracheal tube compared to the conventional tube. Sample size was calculated to detect this difference between the two groups using a two-sided Chi-square test. Therefore, a minimum of 43 patients was required in each group (86 patients in total) with a power of 0.8 and a type I error of 0.05.

Data were presented as mean (SD), median (25th percentile, 75th percentile), or number of patients (%), as appropriate. Data distribution normality was tested using the Kolmogorov-Smirnov test. The rate of endotracheal tube tip at the center of laryngeal inlet and the first attempt success rate were compared by Chi-square test. Total endotracheal tube handling time between groups were compared by Student’s *t*-test. Correlations between patient weight and total endotracheal tube handling time or tube delivering time were evaluated using Pearson’s correlation coefficient. All analyses were performed according to intention-to-treat using SPSS software (version 21.0, SPSS Inc., Chicago, IL).

### Trial registration

Clinical Research Information Service (https://cris.nih.go.kr/). Identifier: KCT0003317 (Registered on 2 November 2018).

## Results

**Figure 3:**
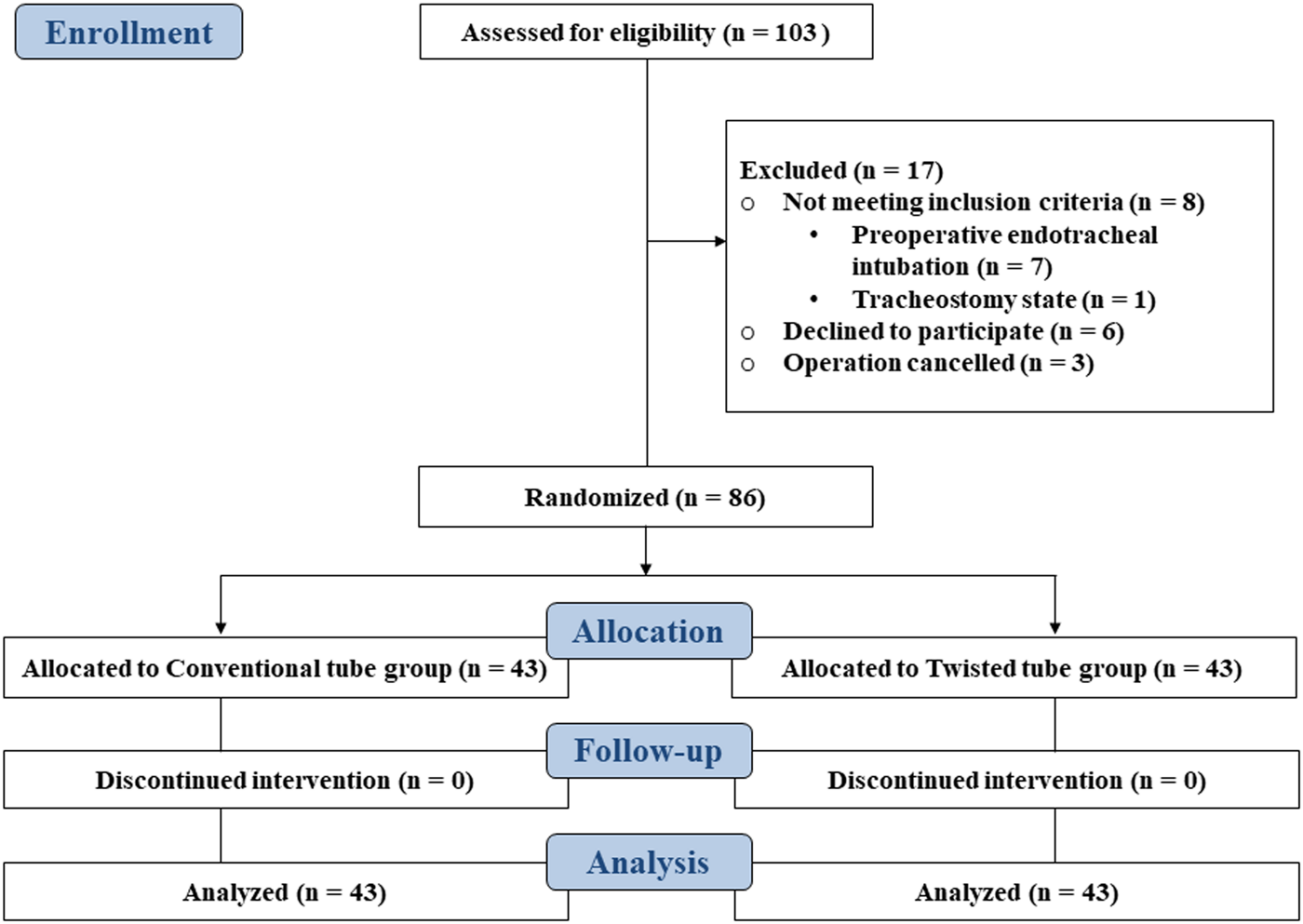
CONSORT Flow Diagram.

Of the 103 patients who were screened for eligibility, 17 were excluded because they did not meet inclusion criteria or declined to participate in the study (Fig. 3). A total of 86 patients completed the study (43 patients in each group). The median age of the study population was 64 (20, 150) days and the median body weight was 4.8 (3.4, 6.9) kg. Baseline data including patient characteristics and intubation tool were comparable between the two groups (Table 1). None of the infants had mouth opening limitations or difficulty in neck extension.

**Table 1:** Baseline characteristics of the patients and the intubation tools.

	Group C (*n* = 43)	Group T (*n* = 43)	*P*-value
Age (day)	60 (16, 120)	67 (21, 210)	0.295
Body weight (kg)	4.5 (3.2, 6.2)	4.9 (3.7, 7.8)	0.150
Body mass index (kg/m²)	15.7 (13.8, 16.7)	15.5 (14.0, 18.4)	0.474
ASA classification (1/2/3/4)	11/19/11/2	13/22/7/1	0.238
Tracheal tube size (3.0/3.5/4.0)	16/25/2	15/23/5	0.577
GlideScope Blade No. (#0/#1/#2/2.5)	0/15/28/0	1/14/27/1	1.000

Values are presented as median (25th percentile, 75th percentile) or frequency.

Intubation data comparing the two groups are presented in Table 2. The initial center location rate for the tube tip at the laryngeal inlet was significantly higher in group T than in group C (88% [38/43] *vs*. 47% [20/43], *P* < 0.001). In patients whose tube tip was not initially centered at the laryngeal inlet, the initial tube tip position was most frequently seen at the right side of the laryngeal inlet, followed by posterior and anterior positions to the laryngeal inlet (Table 2). In addition, total tube handling time was significantly shorter in group T than in group C (15.4 ± 4.7 *vs*. 18.2 ± 5.3, *P* = 0.012). Among the two components of the tube handling time, a significant difference was found in tube delivering time (9.2 ± 3.3 in group T *vs*. 12.7 ± 4.6 in group C, *P* < 0.001) while tube advancement time was comparable between the two groups (Table 2).

**Table 2:** Intubation data in two groups.

	Group C (*n* = 43)	Group T (*n* = 43)	*P*-value
**Initial tube tip location (laryngeal inlet)**			
Center	20/43 (47%)	38/42 (88%)	<0.001
Others (Right/Anterior/Posterior)	13/1/9	4/0/1	
**Total tube handling time, sec**	18.2 ± 5.3	15.4 ± 4.7	0.012
Tube delivering time, sec	12.7 ± 4.6	9.2 ± 3.3	<0.001
Tube advancement time, sec	5.6 ± 2.2	6.3 ± 3.3	0.237
**First attempt success for intubation**	42/43 (98%)	43/43 (100%)	1.000

Values are presented as mean ± Standard deviation or frequency (percent).

Overall, the first attempt success rate was 99% (85/86), and there was no difference between the two groups (Table 2). Patient weight was negatively correlated with total tube handling time (Pearson’s r = −0.286, *P* = 0.008) and tube delivering time (r = −0.276, *P* = 0.01). There were no complications observed after intubation.

## Discussion

In this prospective, randomized, assessor-blinded clinical trial, the spiral shape twist tube effectively facilitated tracheal tube delivery to the center of the laryngeal inlet when compared to the conventional hockey stick shape tube during GlideScope intubation in infants.

Along with the small size of infants, several anatomical features of the airway in infants make intubation procedures using either direct or indirect laryngoscopy more difficult. The larynx is placed further cephalad, which creates a more severe angle between the base of the tongue and the laryngeal inlet. They also have a relatively large tongue, making it difficult to manipulate the endotracheal tube within the small oral cavity^18^. Fast and smooth intubation on the first attempt is needed in infants when considering their physiological vulnerability of rapid oxygen desaturation.

The GlideScope has a definite benefit for better visualization of the larynx^4,6,11,14^. However, there remain factors to be optimized for successful GlideScope intubation such as adequate molding of the endotracheal tube with a stylet and operator experience with the device^12^. Despite the improved laryngeal view, previous studies have shown that the GlideScope takes equivalent or even prolonged intubation time than a direct laryngoscope^6,8,9,19^, and the prolonged intubation time is mainly due to prolonged tube delivery to the larynx^11,13^. Therefore, our study aim was to demonstrate the usefulness of the twist tube for easily handling and delivery of the endotracheal tube in infants. The primary outcome was determined as ‘the initial location of endotracheal tube tip at the laryngeal inlet’ to directly demonstrate the improved accuracy of endotracheal tube handling and delivery rather than reduction of intubation time or number of attempts.

The unique design of the GlideScope blade may be one reason for extended tube delivery time. The tall flange of a Macintosh blade or a Miller blade enables the sweeping of the tongue leftward, making space for visualization and tube passage. However, the GlideScope blade has a small flange that cannot be used to sweep the tongue. Instead, it is recommended to approach underneath the tongue through the midline^20^. This permits a full view of the larynx but potentially blocks the endotracheal tube passage. Moreover, in infants, when the midline space from mouth to laryngeal inlet is occupied by the GlideScope blade, the straight way to the laryngeal inlet for tube delivery is further blocked due to the limited space within the oral cavity.

Given that hypoxemia occurs more rapidly after cessation of assisted ventilation in infants, and that the existing guidelines suggest an acceptable apneic time per intubation attempt of 30 seconds among infants, facilitating the tube delivery during GlideScope intubation is clinically important^21–23^. In a previous study, in the absence of sufficient pre-oxygenation, desaturation accelerated after only 6.6 seconds and 10.8 seconds of apnea in a 1-month-old child and a 1-year-old child, respectively^24^. In this study, by twisting the tube into a spiral shape, the tube could pass through the right posterolateral space underneath the tongue helping the tube tip to reach the laryngeal inlet without impinging on any oral structures. The spiral shape and the angle of the tube led the tube tip towards the center of the laryngeal inlet from the posterolateral space without crossing the midline. No additional re-direction of the tube tip was required to avoid obstacles (e.g., tongue and the GlideScope blade), thereby shortening total endotracheal tube handling time compared to the conventional hockey stick-shaped tube.

In addition, the twisted endotracheal tube in this study effectively facilitated the center location of the tube tip at the laryngeal inlet and significantly reduced unnecessary contact between the tube tip and the peri-laryngeal soft tissues compared to the conventional tube (12% *vs*. 53%). In the conventional tube group, the tube tip was not centered in the larynx in more than half of patients and touched the peri-laryngeal site in order of the right, posterior, and anterior sides. Consistent with our data, a previous study reported that soft tissue injury after GlideScope intubation occurred predominantly on the right side of the larynx^14,15^. Although those were not traumatic, any unnecessary soft tissue contact or re-direction process should be minimized as it may cause soft tissue swelling as well as a delay in intubation time.

Intubation using an indirect laryngoscopy such as the GlideScope requires considerable experience to acquire the relevant hand-eye coordination. As the video display of the GlideScope magnifies the larynx, it may not be easy to determine how much a tube needs to move to locate the tube tip at the intended position, especially for inexperienced anesthesiologists. In this respect, the twist tube we proposed would be helpful to initially place the tube tip at the center of the laryngeal inlet, making tube delivery easier and faster for GlideScope intubation in infants.

To best of our knowledge, this study is the first randomized clinical trial to suggest a novel method to reduce endotracheal tube handling time during GlideScope intubation in infants. In our study, experienced anesthesiologists familiar with the twisted tube performed all tracheal intubations and demonstrated a statistically significant reduction in tube handling time between the two groups. The difference in tube handling time between the two groups may be more prominent in pediatric patients with difficult airways and intubations performed by novice anesthesiologists. However, further data are needed to confirm this.

Nevertheless, this study has some limitations. First, the effect of twisting the tube into the spiral shape was expected to be more pronounced in difficult intubation cases or for younger infants requiring a small sized blade (blade # 0 or blade # 1), as reported in previous cases^16^. In this study, however, infants without airway anomalies were recruited, and the proportion of infants less than 3 kilograms was small. That may make it difficult to demonstrate effectiveness sufficiently. To verify the effectiveness of the new technique, it would be appropriate to study normal airway infants with less possibility of intubation failure. Second, because two experienced and certified anesthesiologists performed all intubations, it is difficult to generalize the results of this study to novice anesthesiologists. The reason for limiting the operators to two expert anesthesiologists was to control confounders caused by variable levels of experience. Third, although statistically negligible, age in days was younger among the conventional tube group. This age difference between the two groups may be due to random chance in a finite sample. Fourth, it is difficult to generalize the results of this study to using intubating stylets with different characteristics, including diameter, flexibility, and the presence of a sheath. In our study, we used an intubating stylet, which was a single-use stylet sheathed in polyvinyl chloride and the external diameter was relatively small compared to the internal diameter of the smallest endotracheal tube used. Thus, the tube advancement time, which refers to the duration for stylet removal, was comparable between the two groups and difficulty removing the stylet was not observed. Finally, performance bias cannot be excluded because it was impossible for operators to be blinded to tube shape during intubation. However, the assessor who analyzed the recorded video remained blinded to group allocation.

## Conclusion

In this prospective, randomized clinical trial, the confined, spiral shape twist tube successfully improved the rate of the initial center location of the tube tip at the laryngeal inlet and facilitated tube delivery in GlideScope intubation in infants. Further studies regarding the efficacy of the spiral shape twist tube in patients with difficult airways are needed.
